# Guide for determination of protein structural ensembles by combining cryo‐EM data with metadynamics

**DOI:** 10.1002/2211-5463.13542

**Published:** 2023-01-09

**Authors:** Z. Faidon Brotzakis

**Affiliations:** ^1^ Department of Chemistry University of Cambridge UK; ^2^ Institute of Bioinnovation BSRC Fleming Vari Greece

**Keywords:** Bayesian inference, cryo‐EM, metadynamics, molecular dynamics, protein dynamics, structural biology

## Abstract

Metadynamics electron microscopy metaInference (MEMMI) is an integrative structural biology method that enables a rapid and accurate characterization of protein structural dynamics at the atomic level and the error in the cryo‐EM experimental data, even in cases where conformations are separated by high energy barriers. It achieves this by incorporating (a) cryo‐electron microscopy electron density maps with (b) metadynamic‐enhanced‐sampling molecular dynamics. Here, I showcase the setup and analysis protocol of MEMMI, used to discover the atomistic structural ensemble and error in the cryo‐EM electron density map of the fuzzy coat of IAPP, a fibril implicated in type II diabetes.

## Graphical abstract

Cryo‐EM has dramatically facilitated the determination of biomolecule structures at the atomic scale. Despite its success, dynamic regions of biomolecules remain poorly resolved. The recently developed metadynamics electron microscopy metaInference (MEMMI) provides a computational approach to accurately and efficiently determine the biomolecular dynamics from cryo‐EM maps. This article provides a step‐by‐step guide to setting up and analysing MEMMI.

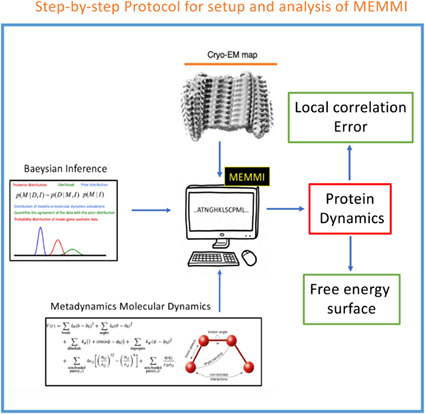

**Abbreviations**
MEMMI: metadynamics electron microscopy metainference
EMMI: electron microscopy metainference
Cryo‐EM: cryo electron microscopy
EMDB: electron microscopy data bank
MD: molecular dynamics
IAPP: islet amyloid polypeptide fibril
PDB: protein data bank
T2D: type‐2 diabetes
GMM: Gaussian mixture model
PBMetaD: parallel‐bias metadynamics

Cryo‐electron microscopy (cryo‐EM) has brought a revolution in structural biology, by giving access to high‐resolution structures of macromolecules in near‐native environments. With more than 20 000 single‐particle electron density maps, the electron microscopy data bank (EMDB) contains rich information about biomacromolecular structures. However, conformational heterogeneity—henceforth referred as structural ensemble—often leads to cryo‐EM density maps representing averaged out structural information [1], leading in turn to low‐resolution regions of the cryo‐EM maps. This effect impedes the reconstruction of an atomistic structure in these regions and leads to structurally missing regions in the final atomistic PDB model.

To address this challenge, inspired by the framework of free energy landscapes and statistical mechanics, the electron microscopy metaInference (EMMI) [2] method models accurately a structural ensemble, by combining noisy (i.e., subject to experimental errors) and heterogeneous (i.e., embedding a structural ensemble) cryo‐EM density maps, with prior knowledge of the system given by molecular dynamics (MD) force fields. Moreover, EMMI can not only characterize the atomistic structural ensemble compatible with the cryo‐EM density map but also the error in the map. So far, EMMI has been employed in various complex biological problems ranging from antibiotic resistance‐related CLP‐protease to microtubule‐tau complexes and SARS‐CoV‐2 membrane receptor proteins and nanobody complexes [3, 4, 5, 6, 7, 8]. However, the accuracy of EMMI depends on the sampling speed.

The recently developed metadynamics EMMI (MEMMI) has enabled to accelerate the generation of atomistic structural ensembles with slowly interconverting states [9]. MEMMI accelerate the structural ensemble sampling by adding a history‐dependent bias to the system as a function of microscopic degrees of freedom of the system known as collective variables (CVs). Albeit MEMMI allows to use many collective variables, thereby reducing the error in made if the choice of CVs is poor, a dedicated review on how to optimize collective variables can be found in Ref. [10]. With this bias, the system is able to escape deep‐free energy minima and transition between structural states. In this protocol, we illustrate a step‐by‐step guide into the preparation and analysis of an MEMMI simulation of our previous study to determine the structural ensemble and cryo‐EM density map error of the full‐length (residue 1–37) islet amyloid polypeptide fibril (IAPP), a notoriously difficult system to characterize atomistically due to the structural heterogeneity and associated errors in the measurement. The formation of IAPP fibrils are associated with the demise of pancreatic β‐cells in type‐2 diabetes (T2D). The low‐density regions of the 12‐residue long N‐terminal tails also known as fuzzy coat are thought to play a key role in amyloid function such as in the binding of RNA, phase separation, and mediation of molecular chaperone binding [11, 12]. Moreover, the fuzzy coat was demonstrated to be involved in membrane binding, either causing catalysis of aggregation directly or by capturing amyloid precursors, consequently facilitating secondary nucleation pathways [11, 12].

## Materials

### Software

1. chimera [13]
2. linux
3. gmconvert [14]
4. rosetta [15]
5. gromacs [16]
6. plumed [17, 18]
7. python [19]
8. mdtraj [20]
9. Access to high‐performance supercomputer

### Cryo‐EM data

**Fig. 1:**
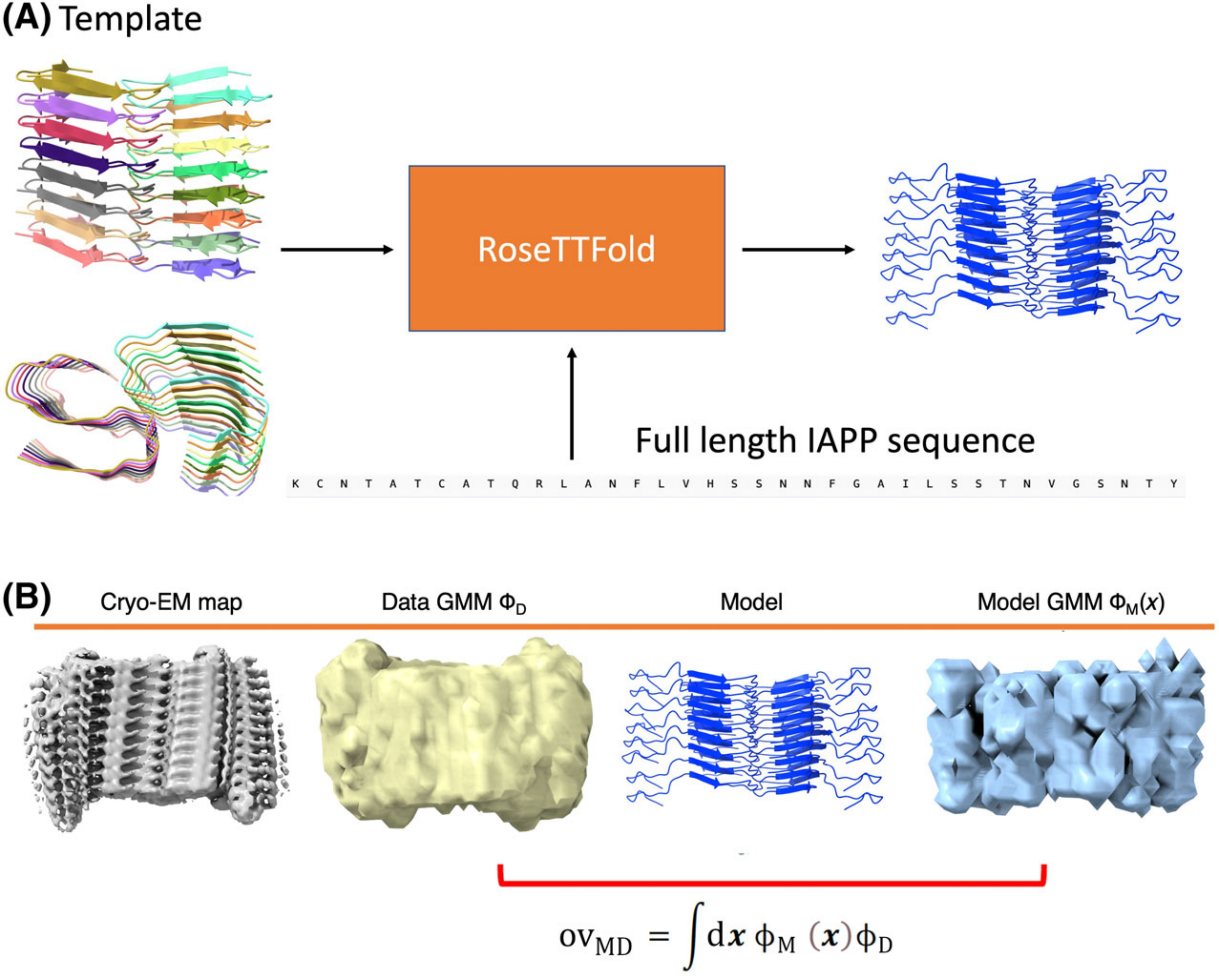
Schematic representation of (A) preparation protocol of the initial full length atomistic model of IAPP. (B) Cryo‐EM density map, data GMM ϕ_D_, containing 200 Gaussians, atomistic model and model GMM ϕ_M_(***x***). The overlap between model and data GMM is shown illustrated with a red bracket.

1. Initial atomic structure of the IAPP fibril (PDB: 6Y1A) [21], see Fig. 1A.
2. IAPP fibril cyo‐EM map EMD‐10669 [21], see Fig. 1B.

## Methods

### Metadynamics electron microscopy MetaInference

While the cryo‐EM density map data consist of voxels on a grid, MEMMI uses a Gaussian mixture model ϕ_D_(***x***) (GMM) conversion of the voxel map, consisting of *N*_D_ Gaussian components

(1) $$\phi_{D}\left(x\right)=\sum_{i=1}^{N_{D}}\phi_{D,i}\left(x\right)=\sum_{i=1}^{N_{D}}\omega_{D,i}G\left(x¦x_{D,i},\sum_{D,i}\right),$$

where ***x*** is a Cartesian vector, ω_D,*i*_ is the scaling factor of the *i*th component of the data GMM, and *G* is a Gaussian around ***x***_D,*i*_ with covariance matrix **∑**_D,*i*_. The overlap function per Gaussian component ov_MD,*i*_

(2) $${\mathrm{ov}}_{\mathrm{MD},i}=\int \phi_{M}\left(x\right)\phi_{D,i}\left(x\right)dx,$$

measures the agreement of MD generated models and the data GMM, where the model GMM ϕ_M_(***x***) is a forward model that converts MD models to GMM. Molecular heterogeneity is dealt in MEMMI by simulating many replicas *r* of the system and hence the overlap in Eqn (2) is estimated over the ensemble of replicas ${\bar{\mathrm{ov}}}_{\mathrm{MD},i}$. In the discrepancy measure, ${\bar{\mathrm{ov}}}_{\mathrm{MD},i}$ is the forward model that is compared to the data‐GMM self‐overlap ${\mathrm{ov}}_{\mathrm{DD},i}=\int \phi_{D}\left(x\right)\phi_{D,i}\left(x\right)dx$ (Fig. 1B).

MEMMI is designed to handle systematic errors, e.g., biases in the force field or forward model, random errors (e.g., due to error in cryo‐EM density map), and errors due to the finite size of the ensemble [22]. Models are generated according to the following MEMMI energy function:

(3) $$E_{\text{MEMMI}}\left(X,\sigma \right)=\begin{array}{l} E_{\mathrm{MD}}\left(X\right)+\frac{k_{B}T}{2}\sum_{r,i}^{N_{R},N_{D}}\frac{{\left[{\mathrm{ov}}_{\mathrm{DD},i}-{\bar{\mathrm{ov}}}_{\mathrm{MD},i}\right]}^{2}}{{\left(\sigma_{r,i}^{B}\right)}^{2}+{\left(\sigma_{i}^{\mathrm{SEM}}\right)}^{2}}\\ +\;E_{\sigma }+\sum_{r=1}^{N_{R}}V_{\mathrm{PB}}\left[s\left(X_{r}\right),t\right], \end{array}$$

where ***X*** represents the atomic coordinates of the structural ensemble, comprising individual replicas *X*_*r*_, where *r* = [1, *N*_R_]. *E*_MD_(***X***) is the energy of the MD forcefield, sampled by multi‐replica MD simulations. The second term quantifies the deviation of the structural ensemble with the data GMM map, while properly considering the error associated to the limited number of replicas in the ensemble σ^SEM^ and the random and systematic errors σ^B^ in the prior, forward model, and experiment. Note σ^SEM^ is estimated per data point ($\sigma_{i}^{\mathrm{SEM}}$) and can either be set to a constant or calculated on the fly by window averaging [23]. σ^B^ is estimated per datapoint *i* and replica *r* as $\sigma_{i,r}^{B}$ and can be sampled by Monte Carlo at each time step, or calculated a posteriori. The third term, *E*_σ_, corresponds to the energy associated with the error σ = (σ^B^, σ^SEM^).

(4) $$E_{\sigma }=k_{B}T\sum_{r,i}^{N_{R},N_{D}}-\log\left(\sigma_{r,i}^{B}\right)+\frac{1}{2}\log\left[{\left(\sigma_{r,i}^{B}\right)}^{2}+{\left(\sigma_{i}^{\mathrm{SEM}}\right)}^{2}\right].\;$$

The fourth term represents the metadynamics bias to accelerate the sampling of the structural ensemble. Parallel‐bias metadynamics (PBMetaD) [24] and multiple‐walkers scheme [25] are used. *V*_PB_ is a time‐dependent biasing potential acting on a set of *N*_CV_ collective variables *s*(***X***), which are functions of the coordinates given as

(5) $$V_{\mathrm{PB}}\left(s_{j}\left(X\right),t\right)=-k_{B}T\log\left\{\sum_{j=1}^{N_{\mathrm{CV}}}\exp\left[\frac{V_{G}\left(s_{j}\left(X\right),t\right)}{k_{B}T}\right]\right\}.$$

The ensemble average ${\bar{\mathrm{ov}}}_{\mathrm{MD},i}\left(X\right)$ is properly unbiased by the biasing potential as in standard umbrella‐sampling [26] by

(6) $${\bar{\mathrm{ov}}}_{\mathrm{MD},i}\left(X\right)=\sum_{r=1}^{N_{R}}w\left(X_{r},t\right)\;{\mathrm{ov}}_{\mathrm{MD},i}\;\left(X_{r}\right)=\sum_{r=1}^{N_{R}}\frac{\exp\left\{\frac{V_{\mathrm{PB}}\left[s\left(X_{r}\right),t\right]}{k_{B}T}\right\}}{\sum_{j=1}^{N_{R}}\exp\left\{\frac{V_{\mathrm{PB}}\left[s\left(X_{j}\right),t\right]}{k_{B}T}\right\}}{\mathrm{ov}}_{\mathrm{MD},i}\;\left(X_{r}\right).$$

The relative error σ_*i*_/ov_DD,*i*_ in the experimental data can be calculated a posteriori by reweighting as ensemble average

(7) $${{\bar{\sigma }}_{i}}^{\mathrm{rel}}=\frac{{\mathrm{ov}}_{\mathrm{DD},i}-{\bar{\mathrm{ov}}}_{\mathrm{MD},i}}{\sqrt{2}}.$$

### System preparation

All the below operations are carried out in a linux or MacOS environment except otherwise specified. The simulation input files can be found in the following github page [27].

**Fig. 2:**
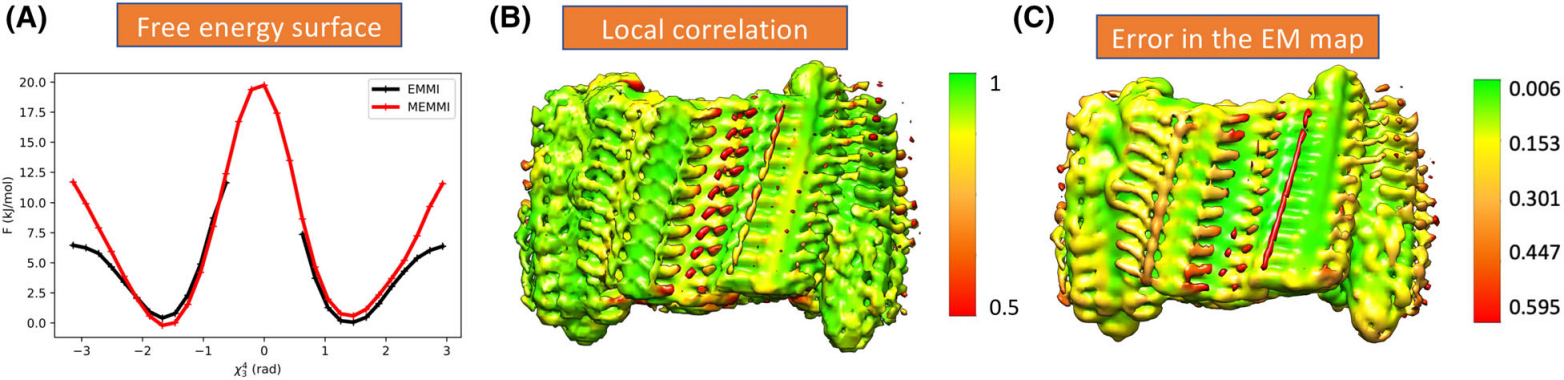
(A) Free energy surface of the disulphide bond CV belonging to the biased and unbiased tail #3 of the peptide [9]. (B) Local correlation between the map and the Cryo‐EM density map (mrc map). (C) Projection in the Cryo‐EM density map of the relative error in the gmm data calculated in box 6 of Analysis section. Figure A has been adapted by using open‐acess data generated in Ref. [9]. [Correction added on 01 February, 2023, after first online publication: Labeling of part A has been corrected in Figure 2]

1. Download and employ PDB 6Y1A as a template in Robetta server and use the full length IAPP sequence as input and the RosettaCM option. This generates a full length atomistic structure models for IAPP (Fig. 1A).
2. Load the atomistic input of step 1. and the EM map EMD‐10669 (cryo_EM_10669.mrc) into chimera (models #0 and #1), respectively, and fit the IAPP structure with the map by executing “*fitmap #0 #1*”. (Fig. 2B). Write a pdb file of this initial IAPP model as iapp_initial.pdb (Fig. 2B third column).
3. Segment the map (model #1) within 6A around the initial model using the command line command “*vop #1 zone #0 6*” followed by saving the segmented map relative to the model #0 to cryo_EM_10669_6A.mrc. This is now considered the experimental voxel cryo‐EM map shown in Fig. 2B first column.
4. Use gmconvert to convert the experimental voxel map generated in the previous step to the data GMM ϕ_D_ by using a divide‐andconquer approach (see Fig. 2B second column). We used the code in Ref. [28, 29] and respective commands in the command‐line are:
python3 generate_gmm.py--input_mapcryo_EM_10669_6A.mrc --n_proc 1
cd ITER_1
cat */*gmm > 1.gmm
gmconvert VcmpG -igmm 1.gmm -imap ../$1 -zth 0.0 -omap iter_1.mrc > iter_1.log
5. Where the last step is a conversion of the data GMM to a readable version for plumed by using “.*/convert_GMM2PLUMED.sh 1.gmm iapp.dat*”, where the file convert_GMM2PLUMED.sh and generate_gmm.py can be found at [29], see Tips and Tricks #1 for the results of this procedure.

### MD equilibration

In a python jupyter notebook, one can use the following commands.

1. Save a no hydrogen structure of the complex.

import mdtraj as md
import numpy as np
from contact_map import ContactMap, ContactFrequency, ContactDifference
import string
import matplotlib.pyplot as plt
import pandas as pd
from collections import defaultdict
iapp=md.load('iapp_initial.pdb')
noh=iapp.topology.select_atom_indices(selection='heavy')
traj_noh=iapp.atom_slice(noh)
traj_noh.save('complex_noh.pdb')

2 Prepare an MD topology by selecting the AMBER99SB‐ILDN [30] and TIP3P [31], respectively. A detailed forcefield parametrization and gromacs setup are highlighted in Tips and Tricks 2. Next, using gromacs, make the simulation box and assess using chimera whether structure.pdb still fits into the cryo‐EM map. Followingly, perform an energy minimization in gromacs.

gmx pdb2gmx ‐f complex_noh.pdb ‐o system.pdb ‐p system.top <<EOF
7
1
EOF
gmx editconf ‐f system.pdb ‐o conf_box.gro ‐c ‐bt triclinic ‐d 1.0
gmx trjconv ‐f conf_box.gro ‐s system.pdb ‐fit translation ‐o conf_box_oriented.gro <<EOF
0
0
EOF
#Asure structure.pdb still fits well the Cryo‐EM map.
gmx trjconv ‐f conf_box_oriented.gro ‐s conf_box_oriented.gro ‐o structure.pdb <<EOF
0
EOF
#Energy minimization
gmx grompp ‐f em.mdp ‐c conf_box_oriented.gro ‐p system.top ‐o em.tpr ‐maxwarn 28
gmx mdrun ‐deffnm em ‐v ‐g

3 Complex solvation, addition of ions, and reminimization of the energy.

gmx solvate ‐cp em.gro ‐cs spc216.gro ‐p system.top ‐o conf_water.gro
gmx grompp ‐f em.mdp ‐c conf_water.gro ‐p system.top ‐o em_solv.tpr ‐maxwarn 28
gmx genion ‐s em_solv.tpr ‐o em_solv_ion.gro ‐p system.top ‐pname NA ‐nname CL ‐neutral <<EOF
13
EOF
gmx grompp ‐f emCG.mdp ‐c em_solv_ion.gro ‐p system.top ‐o em_solv_ion.tpr ‐maxwarn 28
gmx mdrun ‐deffnm em_solv_ion ‐v ‐g

4 Make index groups.

gmx make_ndx ‐f em_solv_ion.gro <<EOF
r GLU ¦ r ASP
a OE1 ¦ a OE2 ¦ a OD1 ¦ a OD2
18 & 19
2 & ! 20
q
EOF

5 Connect to a supercomputer and make a directory. The necessary gromacs molecular dynamics equilibration and MEMMI parameters can be found at Ref. [27] and in Tips and Tricks 2. In the directory, run short molecular NPT equilibration with position restraints at temperature of 300 K and pressure 1 atm, using 256 total number of cores. Input structures and .mdp files are found and restraining setup details can be found in Ref. [27] and Tips and Tricks 3.

gmx_mpi grompp ‐f eqw_npt.mdp ‐c em_solv_ion.gro ‐p system.top ‐o eqw_npt.tpr ‐maxwarn 28 ‐r em_solv_ion.gro
mpirun ‐v ‐np 256 gmx_mpi mdrun ‐deffnm eqw_npt ‐v ‐ntomp

6 Next, perform a short NVT equilibration for 2 ns, using position restraints at *T* = 300 K and *P* = 1 atm. Input structures and .mdp files are found and restraining setup details can be found in Ref. [27] and Tips and Tricks 4.

gmx_mpi grompp ‐f eqw_nvt.mdp ‐c em_solv_ion.gro ‐p system.top ‐o eqw_nvt.tpr ‐maxwarn 28 ‐r em_solv_ion.gro
mpirun ‐v ‐np XX gmx_mpi mdrun ‐deffnm eqw_nvt ‐v ‐ntomp

### Prepare and run MEMMI

1. First, the plumed version containing MEMMI, needs to be downloaded and installed and patched with an MD engine, e.g., gromacs.

git clone https://github.com/tlhr/plumed2.git plumed2
cd plumed2
git checkout emmi‐bias‐sem
DIR=”/work/e658/e658/fb516/SOFTWARE/GROMACS_PLUMED/gromacs‐2019.6”
cmake .. ‐DGMX_FFT_LIBRARY=fftw3 ‐DCMAKE_C_COMPILER=cc ‐DCMAKE_CXX_COMPILER=CC ‐DGMX_MPI=on ‐DCMAKE_SKIP_RPATH=OFF ‐DCMAKE_INSTALL_PREFIX=$DIR ‐DGMX_SIMD=AVX_256 ‐DFFTWF_LIBRARY=/opt/cray/fftw/3.3.4.11/ivybridge/lib/libfftw3f.so ‐DFFTWF_INCLUDE_DIR=/opt/cray/fftw/3.3.4.11/ivybridge/include/fftw3.h
make ‐j8
cd ../
plumed ‐‐no‐mpi patch ‐p ‐‐shared
cd build_mpi
make ‐j8 install

2 The MEMMI simulation can now be submitted. To do this, the initial structures need to be prepared for each replica and the associated replica folders “r$i” where each replica will run. Below, we perform MEMMI using eight replicas.

nreplica=8
for (( c=0; c<$nreplica; c++));do
timee=$(echo $c*240¦bc ‐l)
echo $c $timee
gmx_mpi trjconv ‐f eqw_nvt.xtc ‐s eqw_nvt.tpr ‐o conf_$c.gro ‐b $timee ‐e $timee <<EOF
0
EOF
gmx_mpi grompp ‐c conf_"$c".gro ‐f nvt_prod.mdp ‐p system.top ‐o topol"$c".tpr ‐maxwarn 5
done
done
for (( c=0; c<$nreplica; c++));do
echo r$c
mkdir r$c
cp plumed.dat r$c/
cp topol"$c".tpr r$c/topol.tpr
done

3 The final command to run MEMMI is the following and needs to be submitted within a submission script in a supercomputer.

mpirun ‐v ‐np XX gmx_mpi mdrun ‐deffnm topol ‐multidir r{0..8} ‐plumed ../plumed.dat ‐v ‐ntomp

4 The MEMMI plumed file used can be found in Ref. [27] and is listed in the box below, where its key details are reported in Tips and Tricks 5. The index group contains all protein atoms except for hydrogens and ASP or GLU side chain. This index atom groups are employed to prepare the model gmm δ_M_(***x***) (see Fig. 2B fourth column for a schematic example) to be compared with the overlap function with the data GMM δ_D_. on the fly. The “WHOLEMOLECULES” flag aligns the MEMMI models to the map before comparison. The ENTITY flag specifies the atoms spanning each iapp peptide chain and the respective. RESOLUTION is the cryo‐EM map resolution and the NOISETYPE is the way to determine the error in the data and here we use a Gaussian noise. Output are written every 500 MD steps.

MOLINFO STRUCTURE=../structure.pdb
# define all heavy atoms using GROMACS index file
protein‐h: GROUP NDX_FILE=../index.ndx NDX_GROUP=Protein‐H
protein: GROUP NDX_FILE=../index.ndx NDX_GROUP=Protein
system: GROUP NDX_FILE=../index.ndx NDX_GROUP=System
# make protein whole: add reference position of first heavy atom (in nm)
WHOLEMOLECULES ENTITY0=1‐537 ENTITY1=538‐1074 ENTITY2=1075‐1611 ENTITY3=1612‐2148 ENTITY4=2149‐2685 ENTITY5=2686‐3222 ENTITY6=3223‐3759 ENTITY7=3760‐4296 ENTITY8=4297‐4833 ENTITY9=4834‐5370 ENTITY10=5371‐5907 ENTITY11=5908‐6444 ENTITY12=6445‐6981 ENTITY13=6982‐7518 ENTITY14=7519‐8055 ENTITY15=8056‐8592 ADDREFERENCE
# Constrain the protein CA protofibril core
RMSD REFERENCE=../fibril_core.pdb TYPE=OPTIMAL LABEL=rmsd
UPPER_WALLS ARG=rmsd AT=0.3 KAPPA=0.0 EXP=3 OFFSET=0 LABEL=rmsdwall
# Disulfide dihedral (CYS2:CB,CYS2:S,CYS7:S,CYS7:CB)
disulf1: TORSION ATOMS=29,32,94,91
disulf2: TORSION ATOMS=1103,1106,1168,1165
disulf3: TORSION ATOMS=2177,2180,2242,2239
disulf4: TORSION ATOMS=3251,3254,3316,3313
disulf5: TORSION ATOMS=4325,4328,4390,4387
disulf6: TORSION ATOMS=5399,5402,5464,5461
disulf7: TORSION ATOMS=6473,6476,6538,6535
disulf8: TORSION ATOMS=7547,7550,7612,7609
# Tail definitions
tail1: GROUP NDX_FILE=../index.ndx NDX_GROUP=tail1
tail3: GROUP NDX_FILE=../index.ndx NDX_GROUP=tail3
tail5: GROUP NDX_FILE=../index.ndx NDX_GROUP=tail5
tail7: GROUP NDX_FILE=../index.ndx NDX_GROUP=tail7
tail9: GROUP NDX_FILE=../index.ndx NDX_GROUP=tail9
tail11: GROUP NDX_FILE=../index.ndx NDX_GROUP=tail11
tail13: GROUP NDX_FILE=../index.ndx NDX_GROUP=tail13
tail15: GROUP NDX_FILE=../index.ndx NDX_GROUP=tail15
# Inter‐tail contacts
con1: COORDINATION GROUPA=tail1 GROUPB=tail3 R_0=0.8
con2: COORDINATION GROUPA=tail3 GROUPB=tail5 R_0=0.8
con3: COORDINATION GROUPA=tail5 GROUPB=tail7 R_0=0.8
con4: COORDINATION GROUPA=tail7 GROUPB=tail9 R_0=0.8
con5: COORDINATION GROUPA=tail9 GROUPB=tail11 R_0=0.8
con6: COORDINATION GROUPA=tail11 GROUPB=tail13 R_0=0.8
con7: COORDINATION GROUPA=tail13 GROUPB=tail15 R_0=0.8
# COM for torsions later
com‐cys2‐1: COM ATOMS=@back‐2
com‐cys7‐1: COM ATOMS=@back‐7
com‐as14‐1: COM ATOMS=@back‐14
com‐as31‐1: COM ATOMS=@back‐31
com‐cys2‐2: COM ATOMS=@back‐76
com‐cys7‐2: COM ATOMS=@back‐81
com‐as14‐2: COM ATOMS=@back‐88
com‐as31‐2: COM ATOMS=@back‐105
com‐cys2‐3: COM ATOMS=@back‐150
com‐cys7‐3: COM ATOMS=@back‐155
com‐as14‐3: COM ATOMS=@back‐162
com‐as31‐3: COM ATOMS=@back‐179
com‐cys2‐4: COM ATOMS=@back‐224
com‐cys7‐4: COM ATOMS=@back‐229
com‐as14‐4: COM ATOMS=@back‐236
com‐as31‐4: COM ATOMS=@back‐253
com‐cys2‐5: COM ATOMS=@back‐298
com‐cys7‐5: COM ATOMS=@back‐303
com‐as14‐5: COM ATOMS=@back‐310
com‐as31‐5: COM ATOMS=@back‐327
com‐cys2‐6: COM ATOMS=@back‐372
com‐cys7‐6: COM ATOMS=@back‐377
com‐as14‐6: COM ATOMS=@back‐384
com‐as31‐6: COM ATOMS=@back‐401
com‐cys2‐7: COM ATOMS=@back‐446
com‐cys7‐7: COM ATOMS=@back‐451
com‐as14‐7: COM ATOMS=@back‐458
com‐as31‐7: COM ATOMS=@back‐475
com‐cys2‐8: COM ATOMS=@back‐520
com‐cys7‐8: COM ATOMS=@back‐525
com‐as14‐8: COM ATOMS=@back‐532
com‐as31‐8: COM ATOMS=@back‐549
# Tail torsion (CYS2:CA,CYS7:CA,ASN14:CA,ASN31:CA)
tor1: TORSION ATOMS=com‐cys2‐1,com‐cys7‐1,com‐as14‐1,com‐as31‐1
tor2: TORSION ATOMS=com‐cys2‐2,com‐cys7‐2,com‐as14‐2,com‐as31‐2
tor3: TORSION ATOMS=com‐cys2‐3,com‐cys7‐3,com‐as14‐3,com‐as31‐3
tor4: TORSION ATOMS=com‐cys2‐4,com‐cys7‐4,com‐as14‐4,com‐as31‐4
tor5: TORSION ATOMS=com‐cys2‐5,com‐cys7‐5,com‐as14‐5,com‐as31‐5
tor6: TORSION ATOMS=com‐cys2‐6,com‐cys7‐6,com‐as14‐6,com‐as31‐6
tor7: TORSION ATOMS=com‐cys2‐7,com‐cys7‐7,com‐as14‐7,com‐as31‐7
tor8: TORSION ATOMS=com‐cys2‐8,com‐cys7‐8,com‐as14‐8,com‐as31‐8
# Metad bias
PBMETAD …
BIASFACTOR=56 HEIGHT=0.3 PACE=200 WALKERS_MPI LABEL=pb GRID_WSTRIDE=10000 SIGMA=1000 ADAPTIVE=DIFF
ARG=disulf1,disulf2,disulf3,disulf4,disulf5,disulf6,disulf7,disulf8,tor1,tor2,tor3,tor4,tor5,tor6,tor7,tor8,con1,con2,con3,con4,con5,con6,con7
SIGMA_MIN=0.06,0.06,0.06,0.06,0.06,0.06,0.06,0.06,0.06,0.06,0.06,0.06,0.06,0.06,0.06,0.06,0.5,0.5,0.5,0.5,0.5,0.5,0.5
SIGMA_MAX=0.6,0.6,0.6,0.6,0.6,0.6,0.6,0.6,0.6,0.6,0.6,0.6,0.6,0.6,0.6,0.6,20,20,20,20,20,20,20
GRID_MIN=‐pi,‐pi,‐pi,‐pi,‐pi,‐pi,‐pi,‐pi,‐pi,‐pi,‐pi,‐pi,‐pi,‐pi,‐pi,‐pi,0,0,0,0,0,0,0
GRID_MAX=pi,pi,pi,pi,pi,pi,pi,pi,pi,pi,pi,pi,pi,pi,pi,pi,3820,3820,3820,3820,3820,3820,3820
FILE=../HILLS_disulf1,../HILLS_disulf2,../HILLS_disulf3,../HILLS_disulf4,../HILLS_disulf5,../HILLS_disulf6,../HILLS_disulf7,../HILLS_disulf8,../HILLS_tor1,../HILLS_tor2,../HILLS_tor3,../HILLS_tor4,../HILLS_tor5,../HILLS_tor6,../HILLS_tor7,../HILLS_tor8,../HILLS_con1,../HILLS_con2,../HILLS_con3,../HILLS_con4,../HILLS_con5,../HILLS_con6,../HILLS_con7
GRID_RFILES=../GRID_disulf1,../GRID_disulf2,../GRID_disulf3,../GRID_disulf4,../GRID_disulf5,../GRID_disulf6,../GRID_disulf7,../GRID_disulf8,../GRID_tor1,../GRID_tor2,../GRID_tor3,../GRID_tor4,../GRID_tor5,../GRID_tor6,../GRID_tor7,../GRID_tor8,../GRID_con1,../GRID_con2,../GRID_con3,../GRID_con4,../GRID_con5,../GRID_con6,../GRID_con7
GRID_WFILES=../GRID_disulf1,../GRID_disulf2,../GRID_disulf3,../GRID_disulf4,../GRID_disulf5,../GRID_disulf6,../GRID_disulf7,../GRID_disulf8,../GRID_tor1,../GRID_tor2,../GRID_tor3,../GRID_tor4,../GRID_tor5,../GRID_tor6,../GRID_tor7,../GRID_tor8,../GRID_con1,../GRID_con2,../GRID_con3,../GRID_con4,../GRID_con5,../GRID_con6,../GRID_con7
…
EMMI …
ARG=pb.bias REWEIGHT LABEL=gmm NOPBC TEMP=310.0 NL_STRIDE=100 NL_CUTOFF=0.01 ATOMS=protein‐h GMM_FILE=../iapp.dat SIGMA0=5.0 SIGMA_MIN=0.05 DSIGMA=0.1 RESOLUTION=0.42 NOISETYPE=GAUSS OPTSIGMAMEAN SIGMA_MEAN0=2.0 AVERAGING=200 WRITE_STRIDE=10000
…
# translate into bias
emr: BIASVALUE ARG=gmm.scoreb STRIDE=2
# print useful info to file
PRINT ARG=gmm.* FILE=EMMI STRIDE=500
PRINT ARG=disulf1,disulf2,disulf3,disulf4,disulf5,disulf6,disulf7,disulf8,tor1,tor2,tor3,tor4,tor5,tor6,tor7,tor8,con1,con2,con3,con4,con5,con6,con7,pb.bias FILE=COLVAR STRIDE=500

### Analysis

This subsection is dedicated to the analysis of the outcomes. In particular, 1–6 focus, respectively, on how to (1) reconstruct a continuous trajectory of the structural ensemble, (2) plot free energies as a function of collective variables, (3–5) calculate the global–local correlation of the structural ensemble generated EM map with the experimental EM map and (6) Calculate the error in the experimental EM map.

1. For the full analysis one can visit the *MEMMI_iapp_example.ipynb* in Ref. [27] Using gromacs, we first concatenate the trajectories into a single structural ensemble (traj.xtc) file and then recreate the weight of each configuration using the plumed_analysis.dat.

#!/bin/bash
for ((c=0;c<$nrep;c++));do
cd r$c
for part in $(ls *xtc);do
gmx_mpi trjconv ‐f $part ‐s *tpr ‐o nosolv_$part <<EOF
1
EOF
done
gmx_mpi trjcat ‐f "nosolv_"$part ‐o "r"$c"_cat.xtc" <<EOF
0
EOF
cd ‐
done
gmx_mpi trjcat ‐f r0/r0_cat.xtc r1/r1_cat.xtc r2/r2_cat.xtc r3/r3_cat.xtc r4/r4_cat.xtc r5/r5_cat.xtc r6/r6_cat.xtc r7/r7_cat.xtc‐cat ‐o cat_trjcat.xtc ‐settime <<EOF
0
c
c
c
c
c
c
c
EOF
gmx_mpi trjconv ‐f cat_trjcat.xtc ‐s r0/topol.tpr ‐o traj.xtc ‐pbc mol <<EOF
1
EOF
plumed driver ‐‐plumed plumed_analysis.dat ‐‐mf_xtc traj.xtc
plumed driver ‐‐plumed plumed_analysis_unbiased.dat ‐‐mf_xtc traj.xtc
plumed ‐‐no‐mpi driver ‐‐plumed reconstruct.dat ‐‐mf_xtc traj.xtc ‐‐timestep 10
gmx_mpi trjconv ‐f cat_traj_recon.gro ‐s cat_traj_recon.gro ‐o cat_traj_recon_noh.gro

2 To plot the free energy surface of a collective variable, e.g., disulf3 of the 3rd tail of the biased and unbiased side [9], the following commands are executed in the notebook (Fig. 2A):

time=np.loadtxt("FULLBIAS")[:, 0]
bias=np.loadtxt("FULLBIAS")[:, 1]
KBT = 2.49
weights = np.exp(bias/ KBT)
weights /= weights.sum()
disulf4=np.loadtxt("COLVAR")[:, 4]
disulf4_un=np.loadtxt("COLVAR_UNBIASED")[:, 4]
eq_steps=20000
value5, bins5=np.histogram(disulf4[eq_steps:‐1], bins=30, weights=weights[eq_steps:‐1], density=True)
value5_un, bins5_un=np.histogram(disulf4_un[eq_steps:‐1], bins=30, density=True)
logvalue5=‐np.log(value5)
logvalue5_un=‐np.log(value5_un)
plt.plot(bins5_un[:‐1],logvalue5_un,'tab:blue',linewidth=3)
plt.plot(bins5[:‐1],logvalue5,'tab:red',linewidth=3)
plt.legend( ['EMMI','MEMMI'])
plt.xlabel('$\chi_{3}^{4}$ (rad)' )
plt.ylabel('F (kJ/mol)' )
plt.savefig('Disulf4_FESEMMI_METAD.svg',dpi=400,transparent=True, bbox_inches='tight')

3 To calculate the local correlation of the structural ensemble with the cryo‐EM map, first resample the structural ensemble (conf‐protein.pdb) based on weights:

KBT = 2.494339 # kJ/mol, 300K
n_replica = 1
top_file = "structure_noh.pdb"
biased_cvs = {}
nframes = {weights.shape[0]}
traj = md.load("cat_traj_recon_noh.gro")
N = 10000
n_frames = traj.n_frames
inds = np.arange(n_frames)
top = md.load_topology(top_file)
ca_inds = top.select("name CA")
protein_inds= top.select("protein")
(f"resample").mkdir(exist_ok=True)
inds_sample = np.random.choice(inds, size=N, replace=False,
p=weights / weights.sum())
traj[inds_sample].atom_slice(protein_inds).save_pdb((f"resample" / f"conf‐protein.pdb").as_posix())

4 And then use gmconvert to create a structural ensemble based voxel map (MEMMI_ensemble.map)

# Generate a strctural ensemble‐based map using gmconvert.
gmx trjconv ‐f conf‐protein.pdb ‐s conf‐protein.pdb ‐o .pdb
gmconvert ‐ipdb 0.pdb ‐pdbl pdblist ‐omap MEMMI_ensemble.map ‐a2v R ‐gw 0.5
# where pdblist contains:
#0.pdb 1.0
#1.pdb 1.0
#2.pdb 1.0
#…

5 And then use chimera to calculate the local correlation (Fig. 2B) to create a structural ensemble‐based voxel map (MEMMI_ensemble.map)

### Calculate the local correlation in chimera to produce Fig 2.B
1. open emd_10669_6A.mrc
2. Open MEMMI_ensemble.map
3. vop localCorrelation #0 #1 windowSize 5 model #3
4. scolor #0 volume #3 cmap .5,blue:0.75,white:1,red
5. transparency 30 #1
6 colorkey 0,0 0.2,0.05 0.5 blue 0.75 white 1 red
7. Contour level=0.263

6 Finally to calculate the relative error in each Gaussian datapoint of the GMM map we perform the following analysis as a postprocessing step and stored in the pdf_relerror.pdf. and can be projected on the GMM map as shown in Fig. 2C.

import os
from glob import glob
filenames=glob("OV_FILE*")
newdf=[]
df0=[]
df1=[]
k=0
ov_dd=read_plumed(file='OV_FILE.dat‐1',columns=['Data'],step=1)['Data'].to_list()
for file in filenames:
if (k==0):
df=read_plumed(file=file,columns=['ModelScaled'],step=1)
df0=df['ModelScaled'].to_list()
if (k==1):
df=read_plumed(file=file,columns=['ModelScaled'],step=1)
df1=df['ModelScaled'].to_list()
newdf= np.vstack((df0,df1))
#print(len(df),file,len(newdf))
if (k>1):
dfa=read_plumed(file=file,columns=['ModelScaled'],step=1)
df=dfa['ModelScaled'].to_list()
newdf=np.vstack((newdf,df))
#print(len(df),file,len(newdf),'k>1')
k+=1
error=open("rel_error.dat","w")
errorlist=[]
for i in range(0,len(ov_dd)):
ov_md=newdf[:,i].mean()
if (ov_dd[i]>0):
error.write("%s %.4f %.4f %.4f\n" % (i,np.abs((1‐(ov_md/ov_dd[i]))/np.sqrt(2)),ov_md,ov_dd[i]))
errorlist.append(np.abs((1‐(ov_md/ov_dd[i]))/np.sqrt(2)))
error.close()
counts, bins=np.histogram(errorlist, bins=30, density=False)
plt.hist(bins[:‐1], bins, weights=counts,color='red',linewidth=1)
plt.ylabel('Counts' )
plt.xlabel('σ$_r$' )
plt.savefig("pdf_relerror.pdf", bbox_inches='tight')

## Tips & tricks

1. In MEMMI, we first expressed the experimental voxel map data as a data GMM containing 10 000 Gaussians in total showing 0.975 correlation to the original voxel experimental map (Fig. 2B second column).
2. LINCS is used for bonds constraints [32], the Lenard Jones interactions are switched off with a cutoff at 1 nm and the long‐range interactions are treated using PME (Fourier spacing of 0.12 and a 1 nm cut‐off for the short‐range electrostatic interactions). Pair lists are evolved every 10 fs with 1 nm cutoff every 2 fs. Leap frog and velocity rescale are used to integrate Newton's equations and temperature coupling [33]. The Parrinello–Rahman barostat [34] is used in the NPT, with a coupling time constant of 1.0 ps.
3. Cα are position restrained in the 500 ps NPT equilibration with a 200 kJ·mol^−1^ nm^‐2^ force constant, while the temperature and pressure are set to 300 K and 1 atm, respectively.
4. In the 2 ns, 300 K, NVT simulation no position restraints are used.
5. Configurations were saved every 10 ps. The cryo‐EM restraint is calculated every two MD steps, employing neighbor lists for comparing overlaps between model and data GMMs, with cutoff equal to 0.01 and update frequency of 100 steps.
6. An interesting future direction is to utilize the analysis protocol of Analysis section to compare for a particular protein, the structural ensemble generated by MEMMI and other complementary methods such as manifoldem [35], cryofold [36], and mdff [37].

## Conflict of interest

The authors declare no conflict of interest.

## Author contributions

ZFB conceived and designed the project, acquired the data, analyzed and interpreted the data, and wrote the paper.

## Data Availability

The respective code to reproduce the analysis described in Analysis section can be found in https://github.com/fbrotzakis/MEMMI.
